# Knowledge, Attitudes, and Perceived Barriers of Healthcare Practitioners Toward Interdisciplinary Pain Treatment Programs in Saudi Arabia: A Cross‐Sectional Survey

**DOI:** 10.1155/prm/7965951

**Published:** 2026-11-05

**Authors:** Hayat Al-Rwitae, Mohammed Jameel

**Affiliations:** ^1^ Physical Medicine and Rehabilitation Department Medical Rehabilitation Hospital Madinah 42316 Saudi Arabia; ^2^ Physical Medicine and Rehabilitation Department King Fahad Medical City Riyadh 11525 Saudi Arabia kfmc.med.sa

**Keywords:** attitudes, barriers, chronic pain, healthcare practitioners, implementation, interdisciplinary pain treatment, knowledge, Saudi Arabia

## Abstract

**Background:**

Chronic pain affects a substantial proportion of the global population and poses significant challenges to health systems. Interdisciplinary pain treatment programs (IPTPs) offer evidence‐based, biopsychosocial approaches to pain management, yet their implementation in Saudi Arabia remains limited.

**Objectives:**

This study aimed to assess healthcare practitioners’ knowledge and attitudes toward IPTPs and to identify perceived barriers and facilitators to their implementation within the Saudi healthcare system.

**Methods:**

A cross‐sectional online survey was conducted among healthcare practitioners from various disciplines and clinical settings in Saudi Arabia. A structured, content‐validated questionnaire assessed demographic characteristics, knowledge of IPTP principles, attitudes toward interdisciplinary approaches, and perceived barriers and facilitators. Descriptive statistics and inferential analyses were performed using SPSS Version 27. Associations between professional characteristics, knowledge, and attitude scores were examined using chi‐square tests and ANOVA, with significance set at *p* < 0.05.

**Results:**

A total of 86 practitioners participated. Overall, respondents demonstrated limited knowledge of IPTP principles, particularly regarding program structure and evidence‐based outcomes. However, the majority expressed positive attitudes toward IPTPs and supported their integration into national pain management pathways. Significant barriers identified included lack of institutional support, insufficient trained staff, limited awareness, and funding constraints. Knowledge and attitude scores varied significantly by professional discipline, years of experience, and prior exposure to interdisciplinary training (*p* < 0.05).

**Conclusion:**

This study highlights critical knowledge gaps and organizational barriers that must be addressed to facilitate IPTP implementation in Saudi Arabia. Targeted educational initiatives, institutional support, and strategic alignment with national health reforms are essential to enable the structured integration of IPTPs into chronic pain services.

## 1. Introduction

Chronic pain is a prevalent and disabling condition affecting 30%–50% of adults globally [1, 2] and a substantial proportion in regional studies [3]. In Saudi Arabia, the prevalence of chronic pain is estimated at approximately 46.4%, representing a substantial health burden at the national level [3]. Chronic pain negatively impacts functional capacity, psychosocial well‐being, work productivity, and quality of life, while contributing to increased healthcare utilization and societal costs [1, 4]. Managing this complex condition requires a shift from a purely biomedical model toward approaches that address its biopsychosocial dimensions [2, 5].

Interdisciplinary pain treatment programs (IPTPs) have emerged as an evidence‐based model for chronic pain management [5–7]. Unlike multidisciplinary approaches, in which health professionals work in parallel, IPTPs involve structured collaboration, joint goal setting, and coordinated multimodal interventions delivered by professionals from multiple disciplines [8–10]. Robust international evidence demonstrates that IPTPs can improve pain outcomes, functional capacity, psychosocial health, and return‐to‐work rates, while reducing long‐term opioid use and healthcare costs [5–7, 11–13]. Despite this, implementation remains limited globally due to systemic, organizational, and financial barriers [6, 14, 15].

In Saudi Arabia, interdisciplinary rehabilitation is primarily offered for neurological and postacute conditions, and there is a lack of dedicated IPTPs for chronic pain [3, 16]. Furthermore, national data assessing healthcare practitioners’ knowledge, attitudes, and perceptions regarding IPTPs are scarce. This gap poses a challenge to policy development and service planning at a time when the healthcare system is undergoing significant transformation under Vision 2030, which emphasizes integrated, efficient, and patient‐centered care [17–20]. Understanding healthcare practitioners’ perspectives is critical for successful implementation, as they play key roles in identifying operational barriers and enabling systemic changes.

This study aims to assess healthcare practitioners’ knowledge and attitudes toward IPTPs in Saudi Arabia and to identify perceived barriers and facilitators to their implementation. By exploring these perspectives, the findings may inform strategic planning for chronic pain management and support the integration of IPTPs into the national healthcare system.

This study represents the first national assessment of healthcare practitioners’ knowledge, attitudes, and perceived barriers toward IPTPs in Saudi Arabia, providing implementation‐relevant evidence to inform service planning within the context of ongoing health system reforms.

## 2. Methods

### 2.1. Study Design

This study employed a cross‐sectional survey design to assess the knowledge, attitudes, and perceived barriers of healthcare practitioners toward IPTPs in Saudi Arabia. The study followed the methodological principles of scoping and observational research in pain rehabilitation contexts [21–23].

### 2.2. Participants

The target population included healthcare practitioners currently working in various clinical settings across Saudi Arabia, including hospitals, rehabilitation centers, and primary care clinics. Eligible participants were physicians, nurses, physical therapists, occupational therapists, and other allied health professionals involved in the management of patients with chronic pain. A convenience sampling strategy was used to recruit participants through professional networks, institutional mailing lists, and social media platforms. Participation was voluntary, and informed consent was obtained before survey completion.

### 2.3. Instrument

A structured questionnaire was adapted from previously validated tools assessing healthcare providers’ knowledge and attitudes toward pain management and interdisciplinary care [10, 15, 24, 25]. The instrument consisted of four main sections: (1) demographic and professional characteristics; (2) knowledge of interdisciplinary pain management principles; (3) attitudes toward IPTPs; and (4) perceived barriers and facilitators to implementation. Utilizing a systematic and evidence‐based approach [26, 27], the questionnaire underwent face and content validation by a panel of pain specialists, rehabilitation physicians, and methodology experts to ensure clarity and relevance. A pilot test was conducted with 15 practitioners, and minor wording adjustments were made based on their feedback. As the questionnaire was adapted from previously validated tools [10, 15, 24, 25] and underwent formal face and content validation by an expert panel, its validity rests primarily on this content‐validation process; formal internal‐consistency (Cronbach’s alpha) testing was not feasible given the survey structure, which is acknowledged as a limitation.

### 2.4. Data Collection

The online survey was disseminated using secure, web‐based forms. Data collection took place over a three‐month period from October 2024 through December 2024. Participation was anonymous, and respondents could withdraw at any time without consequence. To maximize response rates, reminder messages were sent biweekly during the data collection period.

### 2.5. Data Analysis

All data were analyzed using IBM SPSS Statistics Version 27 (IBM Corp., Armonk, NY, USA). Descriptive statistics (frequencies, percentages, means, and standard deviations) were used to summarize demographic characteristics, knowledge, attitudes, and perceived barriers. Associations between professional characteristics and knowledge or attitude scores were assessed using appropriate inferential statistics, including chi‐square tests for categorical variables and *t*‐tests or ANOVA for continuous variables, depending on data distribution. In addition, multivariable binary logistic regression was performed to identify factors independently associated with knowledge (correct identification of the IPTP definition) and attitudes (perceiving IPTPs as very important), with professional specialty, years of experience, prior awareness of IPTPs, and sex entered as candidate predictors; adjusted odds ratios (aOR) with 95% confidence intervals (CIs) are reported. A significance level of *p* < 0.05 was set for all analyses.

### 2.6. Ethical Considerations

Ethical approval for this study was obtained from the Institutional Review Board of Dammam Medical Complex, Saudi Arabia (IRB number: PHY‐05). Participation was voluntary, and all respondents provided informed consent electronically prior to enrollment. All procedures were conducted in accordance with the ethical principles outlined in the Declaration of Helsinki and relevant national guidelines.

## 3. Results

### 3.1. Participant Characteristics

A total of 86 healthcare practitioners completed the survey. The majority were specialists in Physical Medicine & Rehabilitation (36.3%), followed by physical therapists (24.2%), anesthesiologists (22.0%), and other allied health professionals (12.1%). The “other” category comprised practitioners from family medicine, psychiatry, occupational therapy, and nursing. Most participants had 10 years or more of professional experience (27.5%). Tables 1 and 2 summarize the demographic and professional characteristics of the respondents, as well as the significance between demographics and knowledge/attitude variables.

**TABLE 1:** Demographic and professional characteristics of participants (*n* = 86).

Characteristic	Category	*n* (%)
Age (years)	20–29	8 (8.8)
	30–39	52 (57.1)
	40–49	15 (16.5)
	50–59	9 (9.9)
	60–69	2 (2.2)

Gender	Male	62 (68.1)
	Female	24 (26.4)

Nationality	Saudi	71 (78.0)
	Non‐Saudi	15 (16.5)

Educational level	Bachelor’s	18 (19.8)
	Postgraduate	68 (74.7)

Years of experience	< 1	5 (5.5)
	1–3	16 (17.6)
	3–5	17 (18.7)
	5–10	23 (25.3)
	≥ 10	25 (27.5)

Specialty	Phys. Med & Rehab	33 (36.3)
	Physical Therapy	22 (24.2)
	Anesthesiology	20 (22.0)
	Others	11 (12.1)

Prior pain management experience	Yes	85 (93.4)
	No	6 (6.6)

*Note:* Values are presented as frequency (percentage). Percentages may not sum to 100 due to rounding.

**TABLE 2:** Significant associations between demographics and knowledge/attitude variables.

Variable	*χ* ^2^ value	df	*p* value	Significance
Age	38.208	16	0.001	Significant
Gender	75.923	12	< 0.001	Significant
Specialty	3.843	4	0.428	Not significant
Education	53.415	28	0.003	Significant
Years of experience	31.991	16	0.010	Significant
Prior pain management exposure	43.115	12	< 0.001	Significant

### 3.2. Knowledge of IPTPs

Overall, the respondents demonstrated limited knowledge of IPTP principles. While 75.8% correctly identified the core components of an interdisciplinary program, fewer were able to distinguish it from a multidisciplinary approach. Awareness of the evidence supporting IPTPs in enhancing functional outcomes, improving psychosocial well‐being, and reducing opioid dependence was generally low among respondents [5–7, 12, 13, 21, 28]. Table 3 presents the distribution of knowledge scores across different professional categories.

**TABLE 3:** Knowledge and familiarity with interdisciplinary pain treatment programs (*n* = 86).

Variable	Category	*n* (%)
Familiarity with IPTP	Regular use	17 (18.7)
	Intermittent use	26 (28.6)
	Aware but not implemented	22 (24.2)
	Not aware	21 (23.1)

Correct understanding of IPTP definition	Collaborative biopsychosocial model	69 (75.8)
	Multidisciplinary/non‐collaborative	3 (3.3)
	Not sure	14 (15.4)

Perceived importance of IPTP	Very important	61 (67.0)
	Important	21 (23.1)
	Not sure/unimportant	4 (4.4)

Agreement IPTP benefits patients	Strongly agree	66 (72.5)
	Agree	20 (22.0)
	Neutral/disagree	0

### 3.3. Attitudes Toward IPTPs

Despite limited knowledge, the majority of participants expressed positive attitudes toward IPTPs. A large proportion agreed that interdisciplinary programs can improve patient outcomes and should be integrated into the national health system. However, only 25.3% indicated that they currently work in settings with any structured interdisciplinary collaboration for pain management. Attitude scores were significantly higher among practitioners with postgraduate training in pain or rehabilitation compared to those without such training (*p* < 0.05). Table 4 details attitudes toward IPTPs by professional group.

**TABLE 4:** Participation in interdisciplinary pain treatment programs.

Variable	Category	*n* (%)
Current participation	Yes	23 (25.3)
	No	63 (69.2)

Previous participation	Yes	36 (39.6)
	No	50 (54.9)

Duration of participation	< 1 year	13 (14.3)
	1–3 years	9 (9.9)
	3–5 years	7 (7.7)
	≥ 5 years	9 (9.9)
	Never participated	53 (58.2)

Willingness to attend IPTP training	Yes	84 (92.3)
	No	2 (2.2)

### 3.4. Perceived Barriers and Facilitators

The most frequently cited barriers to implementing IPTPs included lack of institutional support, insufficient trained staff, limited awareness of program structure, and inadequate funding. Organizational and systemic barriers were mentioned more often than individual‐level barriers, reflecting broader structural challenges in the Saudi healthcare system [6, 14–16, 29]. Conversely, key facilitators identified included leadership support, staff training, and clear referral pathways. Tables 5 and 6 summarize the reported barriers and facilitators to IPTP implementation.

**TABLE 5:** Reported barriers to IPTP implementation.

Barrier category	*n* (%)
Care coordination and teamwork challenges	23 (25.3)
Policy, procedure, and administrative gaps	20 (22.0)
Lack of awareness and training	14 (15.4)
Insurance and financial limitations	12 (13.2)
Communication and patient engagement	11 (12.1)
Workforce shortage and infrastructure	11 (12.1)

**TABLE 6:** Reported facilitators for IPTP implementation.

Facilitator category	*n* (%)
Qualified and sufficient workforce	24 (26.4)
Supportive institutional environment	20 (22.0)
Awareness and motivation among providers	15 (16.5)
Financial coverage	12 (13.2)
Interdisciplinary policies	11 (12.1)
Communication and patient engagement	9 (9.9)

### 3.5. Statistical Associations

Chi‐square and ANOVA analyses revealed significant associations between knowledge scores and professional discipline (*p* < 0.01), years of experience (*p* < 0.05), and previous exposure to interdisciplinary training (*p* < 0.05). Positive attitudes were also associated with higher knowledge scores (*p* < 0.01), suggesting that familiarity with IPTP principles may influence openness toward implementation.

To identify factors independently associated with knowledge and attitudes, a multivariable binary logistic regression was additionally performed (Table 7). After adjustment for professional specialty, years of experience, and sex, prior awareness of IPTPs remained independently associated with correct identification of the IPTP definition (aOR 10.6, 95% CI 3.1–37.1; *p* < 0.001). For attitudes, female sex (aOR for male sex 0.16, 95% CI 0.03–0.73; *p* = 0.02) and prior awareness of IPTPs (aOR 4.30, 95% CI 1.33–13.9; *p* = 0.02) were associated with perceiving IPTPs as very important. Given the sample size and the number of outcome events, these adjusted estimates are considered exploratory.

**TABLE 7:** Multivariable logistic regression of factors associated with knowledge and attitudes toward IPTPs (*n* = 86).

Predictor	Adjusted OR	95% CI	*p* value
*Knowledge model: Correct understanding of IPTP definition*			
Specialty: physical therapy (vs PM&R)	1.01	0.22–4.60	0.99
Specialty: anesthesiology (vs PM&R)	0.33	0.07–1.48	0.15
Specialty: other (vs PM&R)	1.22	0.20–7.41	0.83
Experience 3–10 years (vs < 3 years)	0.40	0.09–1.80	0.23
Experience ≥ 10 years (vs < 3 years)	0.76	0.14–4.08	0.75
Prior awareness of IPTP (vs not aware)	**10.64**	**3.05–37.14**	**< 0.001**

*Attitude model: IPTPs perceived as very important*			
Male sex (vs female)	**0.16**	**0.03–0.73**	**0.018**
Specialty: physical therapy (vs PM&R)	0.81	0.22–2.96	0.75
Specialty: anesthesiology (vs PM&R)	1.27	0.31–5.11	0.74
Specialty: other (vs PM&R)	1.56	0.33–7.31	0.58
Experience 3–10 years (vs < 3 years)	1.07	0.30–3.74	0.92
Experience ≥ 10 years (vs < 3 years)	1.12	0.28–4.51	0.87
Prior awareness of IPTP (vs not aware)	**4.30**	**1.33–13.88**	**0.015**

*Note:* Reference categories: PM&R (specialty), < 3 years (experience), female (sex), and not aware of IPTP (prior awareness). Given the limited number of outcome events, estimates are exploratory. Bold denotes *p* < 0.05.

Abbreviations: aOR, adjusted odds ratio; CI, confidence interval; PM&R, physical medicine and rehabilitation.

## 4. Discussion

This national survey sheds light on the current landscape of healthcare practitioners’ knowledge and attitudes, and perceived barriers toward IPTPs in Saudi Arabia. In summary, the findings reveal limited knowledge of IPTP principles, generally positive attitudes toward interdisciplinary approaches, and significant structural and organizational barriers to implementation. These results highlight critical gaps that must be addressed to support the integration of IPTPs into national pain management strategies.

Taken together, the limited knowledge observed among practitioners aligns with findings from previous studies in other regions, where insufficient familiarity with interdisciplinary principles has been documented [10, 15, 24, 25]. Although IPTPs are well established internationally as effective models for managing chronic pain—improving function, psychosocial outcomes, and reducing opioid use [5–7, 12, 13, 28]—many healthcare professionals remain unaware of their structure and evidence base. This lack of knowledge may reflect the current organization of pain services in Saudi Arabia, where interdisciplinary programs are not yet widely available [3, 16]. Notably, pain education at undergraduate and postgraduate levels remains limited, echoing challenges reported in other Middle Eastern contexts [25].

This pattern suggests that positive attitudes can serve as a foundation for targeted interventions. Similar trends have been reported internationally, where healthcare providers often support interdisciplinary collaboration conceptually, even in the absence of direct program experience [14, 15, 30]. These favorable attitudes can serve as a foundation for targeted educational and organizational interventions to promote IPTP adoption. Previous implementation studies have shown that education, leadership engagement, and structured referral systems are critical for successful integration of interdisciplinary pain services [6, 15, 21, 25].

Perceived barriers identified in this study, including lack of institutional support, inadequate staffing, funding constraints, and limited awareness, mirror implementation challenges described globally [6, 14–16, 31]. Organizational and systemic barriers were more frequently reported than individual‐level barriers, indicating that top–down policy and infrastructure changes may be required. Interpreted through the theoretical domains framework (TDF), these barriers correspond principally to the domains of environmental context and resources (workforce, funding, and infrastructure), social and professional role (coordination across disciplines), and knowledge (limited awareness of IPTP structure), while the reported facilitators map to social influences (leadership and institutional support) and skills (staff training); this domain‐level framing provides a theoretical grounding for the identified barriers and helps prioritize targeted implementation strategies. These findings align with Saudi Arabia’s Vision 2030 transformation, which emphasizes integrated, efficient, and patient‐centered care [29]. Leveraging this national framework could provide opportunities to establish IPTPs within existing health system reforms.

The implications of these findings are significant. Addressing the identified gaps will require a multipronged approach: enhancing pain education in undergraduate and postgraduate curricula, introducing targeted continuing professional development programs, and developing institutional policies that support interdisciplinary collaboration. International experiences suggest that system‐level strategies—such as the use of national registries [21, 32], outcome measurement frameworks [33, 34], and guideline‐based approaches [35, 36]—can guide successful implementation.

Strengths of this study include its focus on a previously unexplored topic within Saudi Arabia and the inclusion of a diverse group of healthcare practitioners. However, several limitations should be acknowledged. Multivariable analyses were not performed, which limits adjustment for potential confounding factors and may influence the observed associations. Internal consistency measures (e.g., Cronbach’s alpha) were not formally assessed, which should be considered when interpreting knowledge and attitude scores. The use of convenience sampling may limit generalizability, and self‐reported data are subject to recall and social desirability biases. Additionally, while the survey assessed knowledge and attitudes, it did not evaluate actual clinical behaviors or patient outcomes. Future research should explore these dimensions using longitudinal and interventional designs. Accordingly, the results should be interpreted as exploratory and hypothesis‐generating rather than representative of all healthcare practitioners in Saudi Arabia.

Collectively, this study underscores the need for educational, organizational, and policy interventions to build capacity for interdisciplinary pain care in Saudi Arabia. By addressing knowledge gaps and systemic barriers, healthcare leaders and policymakers can lay the groundwork for sustainable IPTP implementation, improving outcomes for individuals living with chronic pain. These findings are consistent with established implementation concepts, particularly organizational readiness, feasibility, and leadership engagement.

## 5. Conclusion

This study highlights critical gaps in healthcare practitioners’ knowledge of IPTPs in Saudi Arabia, despite generally positive attitudes toward them. The findings underscore that substantial structural and organizational barriers are hindering IPTPs implementation. Targeted education, institutional support, and strategic policy development are needed to integrate IPTPs effectively into chronic pain management, especially in alignment with Saudi Vision 2030. Given the convenience sample, these findings should be interpreted as exploratory and hypothesis‐generating rather than representative of all healthcare practitioners in Saudi Arabia. Future research should explore the study dimensions using longitudinal and interventional designs.

## Author Contributions

Hayat Al‐Rwitae: conceptualization, methodology, data collection, data analysis, and writing–original draft.

Mohammed Jameel: conceptualization, methodology, validation, data collection, and writing–review and editing.

All authors agree to be accountable for the research presented.

## Funding

This research did not receive any specific grant from funding agencies in the public, commercial, or not‐for‐profit sectors.

## Conflicts of Interest

The authors declare no conflicts of interest.

## Data Availability

The data supporting the findings of this study are available from the corresponding author upon reasonable request.
